# Prevalence of swallowing and speech problems in daily life after chemoradiation for head and neck cancer based on cut-off scores of the patient-reported outcome measures SWAL-QOL and SHI

**DOI:** 10.1007/s00405-015-3680-z

**Published:** 2015-06-14

**Authors:** Rico N. Rinkel, Irma M. Verdonck-de Leeuw, Patricia Doornaert, Jan Buter, Remco de Bree, Johannes A. Langendijk, Neil K. Aaronson, C. René Leemans

**Affiliations:** Department of Otolaryngology/Head and Neck Surgery, VU University Medical Center, Amsterdam, The Netherlands; Department of Radiation Oncology, VU University Medical Center, Amsterdam, The Netherlands; Department of Medical Oncology, VU University Medical Center, Amsterdam, The Netherlands; Department of Radiation Oncology, University Medical Center Groningen/University of Groningen, Groningen, The Netherlands; Division of Psychosocial Research and Epidemiology, The Netherlands Cancer Institute, Amsterdam, The Netherlands

**Keywords:** Swallowing, Speech, Patient-reported outcomes, Quality of life, Head and neck cancer, Chemoradiation

## Abstract

The objective of this study is to assess swallowing and speech outcome after chemoradiation therapy for head and neck cancer, based on the patient-reported outcome measures Swallowing Quality of Life Questionnaire (SWAL-QOL) and Speech Handicap Index (SHI), both provided with cut-off scores. This is a cross-sectional study. Department of Otolaryngology/Head and Neck Surgery of a University Medical Center. Sixty patients, 6 months to 5 years after chemoradiation for head and neck squamous cell carcinoma. Swallowing Quality of Life Questionnaire (SWAL-QOL) and SHI, both validated in Dutch and provided with cut-off scores. Associations were tested between the outcome measures and independent variables (age, gender, tumor stage and site, and radiotherapy technique, time since treatment, comorbidity and food intake). Fifty-two patients returned the SWAL-QOL and 47 the SHI (response rate 87 and 78 %, respectively). Swallowing and speech problems were present in 79 and 55 %, respectively. Normal food intake was noticed in 45, 35 % had a soft diet and 20 % tube feeding. Patients with soft diet and tube feeding reported more swallowing problems compared to patients with normal oral intake. Tumor subsite was significantly associated with swallowing outcome (less problems in larynx/hypopharynx compared to oral/oropharynx). Radiation technique was significantly associated with psychosocial speech problems (less problems in patients treated with IMRT). Swallowing and (to a lesser extent) speech problems in daily life are frequently present after chemoradiation therapy for head and neck cancer. Future prospective studies will give more insight into the course of speech and swallowing problems after chemoradiation and into efficacy of new radiation techniques and swallowing and speech rehabilitation programs.

## Introduction

Advanced head and neck cancer is increasingly being treated with organ-preservation protocols such as chemoradiation therapy (CHRT). Organ-preservation protocols aim, next to the foremost goal to cure the disease, also at maintenance of respiration, deglutition, speech, phonation and cosmetics. However, literature reviews revealed that organ-preservation protocols often result in swallowing impairment; also speech problems may occur but these are investigated less often [1–5]. Most of the studies included in these reviews focused on swallowing impairment using videofluoroscopy, fiberoptic endoscopic evaluation of swallowing (FEES™),or toxicity grading protocols, but recent studies involve patient-reported outcomes measures as well [6–10]. Information from objective imaging techniques regarding swallowing impairment is important but does not necessarily relate to patient-reported swallowing outcomes [11–14]. There is broad range of variety of questionnaires used to evaluate swallowing and speech outcomes and the impact on quality of life [2, 15]. The head and neck cancer modules accompanying the EORTC and FACT Quality of Life Questionnaires or the University of Washington Quality of Life Questionnaire are often used. Studies using specific swallowing and speech-specific questionnaires like the MD Anderson Dysphagia Index (MDADI) [16] or the Swallowing Questionnaire on Quality of Life (SWAL-QOL) [17, 18], or the Speech Handicap index (SHI) [19] to assess speech or swallowing problems in daily life after chemoradiation therapy are less often reported.

The goal of the present cross-sectional study was to assess the prevalence of patient-reported speech and swallowing outcome after chemoradiation therapy for head and neck cancer. Measures were chosen that are provided with clear cut-off values: the Dutch versions of the Swallowing Quality of Life Questionnaire (SWAL-QOL) and the Speech Handicap Index (SHI), which enables quantification of patient-reported speech and swallowing problems in daily life [18, 19]. Furthermore, insight will be obtained regarding the association of sociodemographic (age and gender) and clinical factors {comorbidity [Adult Comorbidity Evaluation 27 (ACE-27)] [20], tumor site and stage, radiotherapy scheduling, time since treatment} and food intake (normal, soft diet, or tube feeding) with patient-reported speech and swallowing outcome.

## Materials and methods

### Ethical considerations

The study was conducted according to regular procedures of the local ethical committee of the VU University Medical Center, Amsterdam. All patients provided informed consent.

### Patients

Inclusion criteria comprised primary head and neck carcinoma (all subsites, all stages) and chemoradiation treatment. One hundred and three patients with primary head and neck carcinoma underwent CHRT of which 71 were alive at the time of the study. Seven patients were excluded because of distant metastasis or loco-regional recurrence and 4 patients were excluded because they did not speak Dutch, leaving a study cohort of 60 patients.

Data on age, gender, comorbidity, and tumor and treatment characteristics [site and stage (according to UICC)], radiation technique, time since treatment, placement and removal of gastrostomy tube were collected from the medical records. Comorbidity was assessed with the Adult Comorbidity Evaluation 27 (ACE-27) [20]. The ACE-27 includes 27 comorbid conditions, including cardiovascular, respiratory, gastro-intestinal, renal, endocrine, neurological, immunological, psychiatric and rheumatologic disorders, previous or synchronous malignancy, alcohol abuse and excessive body weight. The ACE-27 was designed specifically for cancer patients and classifies patients into 4 grades of comorbidity [none (grade 0), mild (grade 1), moderate (grade 2), severe (grade 3)].

During a time span of 8 months, 52 out of 60 patients returned the SWAL-QOL (response rate 87 %) and 47, the SHI (response rate 78 %). An overview of patient characteristics is provided in Table 1. Median age of the patients was 58 years (range 36–75). Thirty-five (67 %) of the patients were male. No comorbidity was observed in 16 patients, 24 patients had grade 1, 8 patients grade 2 and 4 patients grade 3. Primary tumor locations were oral cavity (*n* = 5), oropharynx (*n* = 30), nasopharynx (*n* = 4), larynx (*n* = 10) and hypopharynx (*n* = 3) and these were categorized into oral cavity/oropharynx/nasopharynx (*n* = 39) and larynx/hypopharynx (*n* = 13) for comparison and statistical analyses. Overall tumor stages were II (*n* = 2), III (*n* = 18) and IV (*n* = 32), which were categorized into stage II–III (*n* = 20) vs. IV (*n* = 32) for comparison and statistical analyses. Almost all (97 %) patients underwent PEG placement at start of treatment. All patients underwent chemoradiation, in 79 % of the patients consisting of Cisplatin 100 mg/m2 in 3 cycles. Of the remaining patients, 4 % received 1 cycle and 12 % received 2 cycles. Three patients (6 %) underwent intra-arterial Cisplatin 150 mg/m2 in 4 cycles. Chemotherapy was given concomitantly with radiotherapy (2 Gy per fraction, 5 times per week, total dose of 70 Gy). One patient received also 5-Fluor-Uracil because of cisplatin side-effects after 1 cycle. In 31 patients, conventional three-dimensional conformal radiotherapy (3D-CRT) was given and intensity-modulated radiation therapy (IMRT) in 21 patients. Patients treated before 2005 were treated with 3D-CRT, without sparing the salivary glands. In 2005, IMRT was introduced as part of standard care at the department of radiation oncology enabling a significant reduction of the dose to the salivary glands. The gastrostomy tube was removed in 80 % after complete remission and when oral intake was sufficient (in average 3–6 months after end of treatment). Time since treatment ranged from 6 to 58 months with a median of 21 months.

**Table 1:** Overview of patient characteristics (*n* = 52)

Gender	
Male	35 (67 %)
Female	17 (33 %)
Comorbidity	
None	16 (31 %)
Grade 1	24 (46 %)
Grade 2	8 (15 %)
Grade 3	4 (8 %)
Tumor site	
Oral cavity	5 (9 %)
Oropharynx	30 (58 %)
Nasopharynx	4 (8 %)
Larynx	10 (19 %)
Hypopharynx	3 (6 %)
Tumor stage	
II/III	23 (44 %)
IV	29 (56 %)
Radiotherapy	
3D-CRT	31 (60 %)
IMRT	21 (40 %)

### Patient-reported outcomes

Swallowing and speech impairment was measured via the Swallowing Questionnaire on Quality of Life (SWAL-QOL) and the Speech Handicap Index (SHI).

The SWAL-QOL is a 44-item questionnaire on swallowing-related problems in daily life [17, 18]. Response categories range on a 5-point scale. There are 10 subscales: (1) food selection (2 items); (2) eating duration (2 items); (3) eating desire (3 items); (4) Fear (4 items); (5) burden (2 items); (6) mental health (5 items); (7) social functioning (5 items); (8) communication (2 items); (9) sleep (2 items); and (10) fatigue (3 items). Furthermore, there is an overall symptom scale (14 items). Finally, a total SWAL-QOL Score can be calculated (based on the 23 items of the first 7 scales listed above). All SWAL-QOL scales range from 0 to 100, a higher score indicating more impairment. Three single questions are included regarding nutrition intake (“normal”, “soft”, “pureed”, “liquids only”, “mostly tube feeding” and “tube feeding only”), liquid intake (“all liquids”, “thick liquids”, “very thick liquids”, “thickened liquids” and “no liquids”), and general health (“poor”, “moderate”, “good”, “very good” and “excellent”). The SWAL-QOL was translated and validated for use in Dutch head and neck cancer patients. A cut-off score on the total SWAL-QOL score of 14 points (or higher) (94 % sensitivity and 84 % specificity) indicates swallowing problems in daily life [18].

The SHI is a validated speech-specific quality of life questionnaire and consists of 30-items focusing on speech-related problems in daily life. Response categories range on a 5-point Likert scale (“never”, “almost never”, “sometimes”, “almost always” and “always”). The questionnaire also includes an overall speech quality item, with 4 response categories (“good”, “reasonable”, “poor” and “severe”). A total SHI score is calculated by summing the scores on all 30 items (score range 0–120), with a higher score indicating a higher level of speech-related problems. Two subscales are distinguished: psychosocial function and speech function. A total SHI score ≥6 indicates speech problems in daily life (95 % sensitivity and 90 % specificity) [19].

### Statistical analyses

Descriptive statistics were calculated for the SWAL-QOL and SHI scales. A *t* test for independent groups was used to test differences between patients regarding gender (male vs. female), tumor site (larynx/hypopharynx vs. oral cavity/oropharynx/nasopharynx), tumor stage (stage I–II vs. stage III–IV) and type of radiotherapy (3D vs. IMRT) on SWAL-QOL and SHI scores. Analysis of variance (ANOVA) was carried out to test differences regarding comorbidity (grade 0, 1, 2 or 3) and food intake (normal, soft diet, pureed diet, tube feeding). In case of overall significant differences, post hoc analyses were carried out to pairwise compare all different groups. Bonferroni correction was applied to correct for multiple testing. Correlation analyses were performed to study the relation between age (Pearson) and time since treatment (Spearman’s rank) and SWAL-QOL and SHI scores, and to study the relation between the total SWAL-QOL scale and the total SHI scale. Those variables that showed univariate significant relations with the outcome measures, were included into the multivariate regression analysis (stepwise) to obtain insight into which sociodemographic (age, gender) and clinical (comorbidity, tumor site and stage, radiation technique, time since treatment) parameters are associated with patient-reported speech and swallowing outcome. For all statistical tests, significance was defined as *p* < 0.05. All analyses were performed using the IBM Statistical Package for the Social Science (SPSS) version 20 (IBM Corp., Armonk, NY USA).

## Results

### Patient-reported swallowing outcome

A deviant SWAL-QOL score (total SWAL-QOL score ≥14) was observed in 79 % of the patients (41/52). Mean scores are presented in Table 2. Relatively high scores were observed on the subscales general burden, food selection, eating duration, eating desire, fatigue and sleep.

**Table 2:** Summary of mean scores (SD) of all patients on the subscales of the SWAL-QOL and the SHI, and regarding food intake (normal diet vs. soft/pureed vs. tube feeding), and *p* values regarding group differences

	All patients (*n* = 52)	Normal (*n* = 23)	Soft or pureed (*n* = 18)	Tube feeding (*n* = 10)	*p* value			
	Mean (SD)	Mean (SD)	Mean (SD)	Mean (SD)	Overall	Normal vs. soft/pureed*	Normal vs. tube feeding*	Soft/pureed vs. tube feeding*
SWAL-QoL (total score)	**35.8 (23.8)**	**20.0 (19.7)**	**50.6 (17.9)**	**48.5 (17.3)**	<0.001	<0.001	0.001	1.00
Symptomen schaal van de SHI	**35.4 (19.5)**	**23.8 (19.0)**	**43.7 (15.3)**	**49.6 (10.7)**	<0.001	0.001	0.001	1.00
General burden	**42.7 (30.4)**	**25.1 (29.2)**	**60.6 (22.8)**	**52.6 (24.2)**	<0.001	<0.001	0.023	1.00
Food selection	**45.8 (34.0)**	**20.7 (24.6)**	**69.0 (20.1)**	**66.5 (30.0)**	<0.001	<0.001	<0.001	1.00
Eating duration	**56.9 (32.2)**	**38.2 (30.1)**	**73.7 (21.0)**	**72.6 (31.5)**	<0.001	<0.001	0.005	1.00
Eating desire	**39.8 (31.9)**	**19.5 (27.5)**	**51.3 (20.6)**	**69.9 (25.0)**	<0.001	0.001	<0.001	0.19
Fear of eating	**30.2 (24.4)**	**21.8 (22.0)**	**42.6 (22.2)**	**30.1 (25.8)**	0.021	0.017	1.00	0.52
Sleep	49.5 (31.3)	39.8 (29.7)	59.3 (25.6)	53.9 (41.3)	0.13	0.15	0.71	1.00
Fatigue	47.1 (24.3)	43.1 (29.2)	47.7 (17.2)	55.0 (24.3)	0.44	1.00	0.62	1.00
Communication	32.3 (29.2)	20.7 (23.8)	38.3 (30.4)	46.3 (32.4)	0.034	0.15	0.059	1.00
Mental health	**30.6 (26.5)**	**16.1 (19.8)**	**44.4 (22.2)**	**41.0 (31.6)**	0.001	0.001	0.021	1.00
Social FX	**28.8 (25.8)**	**13.5 (17.1)**	**43.3 (26.0)**	**40.5 (22.5)**	<0.001	<0.001	0.006	1.00
SHI (total score)	18.6 (21.8)	11.7 (15.2)	22.8 (27.7)	29.3 (22.6)	0.11	0.38	0.19	1.00
SHI psychosocial subscale	6.7 (10.7)	3.0 (6.4)	10.2 (13.7)	10.3 (11.2)	0.069	0.11	0.33	1.00
SHI speech quality subscale	11.8 (12.0)	8.4 (10.1)	12.7 (13.5)	18.7 (11.8)	0.12	0.84	0.14	0.79

Statistical significant differences (*p* < 0.05) are printed bold

* *p* values are corrected with the Bonferroni correction to account for multiple testing

Normal food intake was reported by 23 patients (45 %), while 18 (35 %) took a soft diet or pureed food, and 10 (20 %) had tube feeding (data were missing for 1 patient). Patients with normal food intake had significantly better mean scores compared to patients with soft diet or tube feeding on all SWAL-QoL scales, except for the subscales sleep and fatigue; patients with soft diet or tube feeding did not differ from each other significantly on any of the scales (Table 2).

Univariate analyses revealed that age, gender, comorbidity and tumor stage were not significantly related to SWAL-QOL scores. Swallowing outcome was significantly associated with tumor site and radiotherapy technique. Patients treated for a laryngeal or hypopharyngeal tumor had significantly (*p* < 0.05) better scores compared to patients treated for an oral cavity, oropharyngeal or nasopharynx tumor on the total SWAL-QOL (*df* = 50; *t* = −2.10; *p* = 0.041) and the subscales general burden (*df* = 50; *t* = *−*2.23; *p* = 0.030), mental health (*df* = 50; *t* = −2.30; *p* = 0.026) and social functioning (*df* = 50; *t* = −2.46; *p* = 0.017). Compared to 3D-CRT, patients after IMRT had significantly better scores on the total SWAL-QOL (*df* *=* 49.9; t = 2.22; *p* = 0.031), food selection (*df* = 50; *t* = 2.01; *p* = 0.05), fear of eating (*df* = 50; *t* = 3.34; *p* = 0.002), sleep (*df* = 50; *t* = 2.89; *p* *=* 0.006) and social functioning (*df* = 50; *t* = 2.46; *p* = 0.018). Furthermore, a positive correlation (more problems on the long term) was found between time since treatment and the following SWAL-QOL subscales:fear of eating (*ρ* = 0.57; *p* < 0.001), sleep (*ρ* = 0.41; *p* = 0.002), fatigue (*ρ* = 0.33; *p* = 0.015), social function (*ρ* = 0.33; *p* = 0.019) and the total SWAL-QOL score (*ρ* = 0.37; *p* = 0.007).Because radiotherapy technique was related to time since treatment, correlation coefficients were also calculated in these subgroups (3D-CRT vs. IMRT) and no significant relations between time since treatment and swallowing outcome were found.

For the multivariable regression analysis only tumor site and radiotherapy technique were included in the selection procedure, which revealed that only tumor site was significantly associated with the total SWAL-QOL score (*B* = 15.5, *R*^2^ = 0.081, *F* = 4.40, *p* = 0.041). Note that radiotherapy technique is univariate more significantly associated to total SWAL-QOL, but this factor did not enter the multivariate model due to unequal variances of the two groups.

### Patient-reported speech outcome

A deviant SHI score (total SHI score ≥6) was observed in 55 % of the patients (26/47). Mean scores are presented in Table 2. Univariate analyses revealed that radiotherapy technique was associated significantly with the subscale SHI psychosocial function: patients treated with IMRT had a better score (*df* = 39.7; *t* = 2.48; *p* = 0.017). Age, gender, comorbidity, tumor stage and site, and time since treatment (corrected for radiotherapy technique) were not significantly associated with SHI total scores. Therefore, no multivariate regression analyses were carried out.

### Relation between swallowing and speech outcome

A significant relation between swallowing and speech outcome was found: Pearson r was 0.56, *p* < 0.001 regarding the total scores on the SWAL-QOL and SHI. Regarding the presence of swallowing and speech problems (score above cut-off value), 51 % of the patients after chemoradiation had both speech and swallowing problems, 24 % had swallowing problems but no speech problems, 7 % had speech problems but no swallowing problems and 18 % had no speech or swallowing problems.

## Discussion

The present study revealed a high prevalence of patient-reported swallowing (79 %) and speech (55 %) problems after chemoradiation for advanced head and neck cancer. Swallowing and speech problems were significantly related to each other, indicating that many patients who experience swallowing problems also experience speech problems. The prevalence of swallowing and speech problems after (chemo)radiotherapy in earlier studies differ considerably depending among others on the assessment methods [2, 15]. Unlike earlier studies, the percentages in the present study are based on validated cut-off values of the SWAL-QOL and SHI questionnaires.

Swallowing problems can lead to clinically apparent as well as silent aspiration or continued alternate feeding such as feeding tube placement [33, 34]. Recently, Kano et al. [35] compared the need for tube feeding support among patients treated with surgery (26 %) vs. chemoradiation (12 %), as assessed immediately after initial treatment. In the present study, 20 % of the patients after CHRT had tube feeding, almost all within 18 months after treatment, which falls in the broad range as reported in the literature varying from 20 to 60 % use of a feeding tube at 1 year after treatment to 8–18 % longer term use [36, 37]. As expected, patients with tube feeding as well as patients with soft or pureed diet in the present study had more swallowing problems compared to patients with normal oral food intake. Dysphagia is known to have a major impact on quality of life, in particular in patients with tube feeding [38–40]. Severe dysphagia is also related to increased emotional distress, not only of the patients themselves but also of their spouses [41, 42].

In the present study, swallowing and speech problems were significantly related to tumor subsite (patients treated for oral or oropharynx cancer had significantly more swallowing problems compared to patients treated for larynx or hypopharynx cancer) and radiation technique (patients treated with IMRT reported less swallowing and speech problems). There are several causes that may explain these differences. Detailed studies on swallowing after (chemo)-radiation for head and neck cancer revealed a large variety of motility disorders, including prolonged oral transit time, decreased tongue strength/control, reduced base of tongue contact to the pharyngeal wall, pharyngeal constrictor dysmotility, decreased laryngeal elevation, reduced hyoid movement and epiglottic dysmotility [21–25]. The probability of swallowing dysfunction after (chemo)radiation appears to be associated with radiation-induced thickening of the pharyngeal constrictor muscles, the supraglottic larynx and the glottic larynx [26, 27]. Furthermore a clear relationship is found between dose distributions in the salivary glands and subjective xerostomia [28, 29]. Chemotherapy functions as a sensitizer for radiotherapy and enhances the effects of radiotherapy on the tumor and the surrounding tissue. The cytotoxic effects of chemotherapy alone on oral, pharyngeal and laryngeal mucosa also lead to oral mucositis, infections, xerostomia, and neutropenia are associated with long-term swallowing problems, not only in head and neck cancer patients [30, 31], but also in, for example, breast, colon or lung cancer patients [32].

The prevalence of swallowing dysfunction and the major impact of this side effect on the more general dimensions of health-related quality of life stresses the importance of effective preventive measures and/or therapeutic interventions. New radiation delivery techniques aiming at sparing of anatomical structures that are correlated with swallowing may contribute to prevent long-term radiation-induced dysphagia [43–45]. Another possibility is speech and swallowing rehabilitation. In usual care, rehabilitation includes pretreatment evaluation of swallowing and counseling allowing to determine the swallowing status at start and to prepare the patient regarding possible swallowing impairment and the rehabilitation process and post treatment speech and swallowing management strategies consisting of oromotor exercises (to increase the strength and mobility of the lips, tongue and mandible), swallow maneuvers (to facilitate swallowing function and to prevent aspiration) and compensation techniques (adjusting posture, adjusting food consistency). It has been argued that patients should be encouraged to swallow throughout their treatment also when prophylactic feeding tube is placed. Recent studies reveal that exercises in an early stage, before and during radiotherapy, may prevent or decrease swallowing dysfunction after curative (chemo)-radiation [5, 46–49], but not all studies show these beneficial effects [50]. There is growing evidence that attention is needed for the individual needs of the patients to determine the best rehabilitation strategy [51, 52]. Prospective randomized trials are needed to provide evidence-based effectiveness of these approaches.

It can be concluded that swallowing and (to a lesser extent) speech problems in daily life are frequently reported by patients after chemoradiation therapy for advanced head and neck cancer. Future prospective studies will give more insight into the course of speech and swallowing problems after chemoradiation and into efficacy of new radiation techniques and swallowing and speech rehabilitation.
